# A trend in prevalence of antimicrobial use and appropriateness of antimicrobial therapy in an acute care hospital from 2018 to 2019: repeated prevalence surveys in Japan

**DOI:** 10.1186/s13104-019-4849-0

**Published:** 2019-12-18

**Authors:** Junpei Komagamine, Taku Yabuki, Taku Hiraiwa

**Affiliations:** Department of Internal Medicine, National Hospital Organization Tochigi Medical Center, 1-10-37, Nakatomatsuri, Utsunomiya, Tochigi Japan

**Keywords:** Antimicrobial stewardship, Antimicrobial therapy, Inpatient antimicrobial use

## Abstract

**Objectives:**

The supply of cefazolin has been reduced dramatically since March 2019 in Japan. However, no studies have evaluated the effects of cefazolin shortage on the appropriateness of antimicrobial use. Therefore, we compared the appropriateness of inpatient antimicrobial drug use between the two surveys conducted in August 2018 and August 2019 in a Japanese hospital with no supply of cefazolin since the cefazolin shortage.

**Results:**

In the 2019 survey, a total of 275 patients were included, and 256 patients were included in the 2018 survey. The prevalence of antimicrobial drug use did not change from 2018 to 2019 (28.5% versus 28.7%; *p *= 0.96). The proportion of cefazolin in all antimicrobial drugs used on the survey date significantly decreased from 2018 to 2019 (24.1% versus 0.0%; *p *< 0.001). The proportion of appropriate antimicrobial use in all antimicrobial therapies tended to be lower in 2019 than in 2018 (41.2% versus 60.6%; *p* = 0.06), and the proportion of unnecessary use of a broader spectrum of antimicrobial drugs significantly increased from 2018 to 2019 (4.7% versus 37.3%; *p* = 0.002). The shortage of cefazolin might lead to inappropriate use of other antimicrobial drugs.

## Introduction

Antimicrobial resistance is a major threat to public health. Infection caused by antimicrobial-resistant bacteria is associated with a poor prognosis and a higher cost burden [1, 2]. One strategy to reduce antimicrobial-resistant bacteria is to improve the appropriateness of antimicrobial drug use [3]. In an inpatient setting, the prevalence of antimicrobial drug use is 30–50% [4–14]. However, approximately one-third of their use is considered unnecessary or inappropriate [12, 15–21].

In Japan, the supply of several antimicrobial drugs, such as ampicillin–sulbactam [22], has decreased for several reasons since 2018. The supply of cefazolin to Japanese hospitals has also been reduced dramatically because Nichi-Iko Pharmaceutical Co., which covered approximately 60% of the domestic market share of cefazolin in Japan, stopped production of cefazolin due to contaminated ingredients since March 2019 [23–25]. Therefore, for the Japanese hospitals that depended only on Nichi-Iko Pharmaceutical Co. for cefazolin, their supply has stopped completely [24]. Given that cefazolin is one of most frequently used parenteral antimicrobial drugs in Japan [26], no supply of cefazolin can dramatically change inpatient antimicrobial use. Moreover, given that the spectrum activity of cefazolin was the narrowest in the top five consumptive parenteral antimicrobial drugs in Japan [26], a shortage of cefazolin may cause the excess use of antimicrobial drugs that have unnecessarily broader spectrum activity. However, no studies have been conducted to evaluate the appropriateness of antimicrobial therapy in Japan since March 2019. Therefore, we conducted a point prevalence survey to investigate the appropriateness of antimicrobial therapy in a Japanese hospital with no supply of cefazolin since March 2019 and compared this outcome with those of the survey conducted in August 2018 [14].

## Main text

### Methods

#### Study design and setting

The National Hospital Organization Tochigi Medical Center is a 350-bed community acute care hospital located in Utsunomiya, Japan. In our hospital, the supply of cefazolin has been suspended completely since March 2019. Since the beginning of 2019, an antibiotic restriction policy for cefazolin has been applied in our hospital. Point prevalence surveys were conducted twice on 6 August 2018 and on 22 August 2019. The results of the former survey were already reported in a previous article [14]. Our hospital had an antimicrobial stewardship team composed of physicians, nurses, pharmacists, and laboratory technicians before the time of these surveys. For each survey, all hospitalized patients at 8:00 A.M. on the survey day were included. No patients were excluded. This study was prospectively registered at the University Hospital Medical Information Network clinical registry (No. UMIN000037720).

#### Data collection and outcome measures

Data were collected in a manner similar to a previous study [14]. Physicians retrieved information on patient age, sex, and past medical history using electronic medical records. Information on the devices in place was collected by infection control nurses during the morning of the survey day. Regarding antimicrobial drugs, drug prescription data from electronic medical records were used. Based on past studies [4, 14], a patient was considered to receive an antimicrobial drug if the patient was prescribed or planned to be prescribed any antimicrobial drug on the survey date or the calendar day before the survey date. Antimicrobial drugs were classified based on the World Health Organization Anatomical Therapeutic Chemical classification. Drugs used to treat HIV or viral hepatitis and topical antimicrobial drugs were excluded. Like the previous study [14], the reasons for use of each antimicrobial drug were abstracted from the electronic medical records and classified into the following five categories: treatment of infection, surgical prophylaxis, medical prophylaxis, a noninfection-related reason, or unknown reason.

The primary outcome was the proportion of antimicrobial drugs that were judged to be appropriately used in all antimicrobial therapies. The appropriateness of antimicrobial therapy was assessed by members of the antimicrobial stewardship teams based on the results of microbial tests at the time of the survey, antibiograms of our hospital, and the Infectious Diseases Society of America Practical Guidelines [27]. The following five points were evaluated for the appropriateness of antimicrobial drugs for infection treatment: (1) indication of the antimicrobial therapy, (2) dose or timing of the antimicrobial drug, (3) duration of the antimicrobial drug use, (4) choice of the antimicrobial drug, and (5) spectrum activity of the antimicrobial drug. Antimicrobial drugs used for infection treatment were judged to be appropriate if the use of antimicrobial drugs was appropriate for all five points. The detailed methods to investigate these outcomes were described in a recent article [14]. The secondary outcomes were the proportion of patients who were received antimicrobial drugs and the proportion of cefazolin among all antimicrobial drugs. These outcomes were compared with those of the survey performed in 2018 [14].

#### Statistical analysis

The study population was described by using descriptive statistics. Fisher’s exact test was used to compare the primary and secondary outcome between the 2018 survey [14] and the 2019 survey. The Stata version 15 (LightStone, Tokyo, Japan) was used for these analyses, and the level of significance was set at 5%.

### Results

In the present survey, a total of 275 hospitalized patients were included, while 256 patients were included in the previous survey. In the present survey, the median patient age was 76 years (IQR 65 to 85), 138 (50.2%) were women, and 23 (8.4%) were immunosuppressive drug users (Table 1). The baseline characteristics of the patients in the present survey were similar to those in the previous survey.

**Table 1:** Clinical and demographic characteristics of surveyed patients according to survey year

Characteristics	August 2018 (n = 256)	August 2019 (n = 275)
Age, year, median (IQR)	76 (67–84)	76 (65–85)
Age category, n (%)		
< 1 year	2 (0.8)	5 (1.8)
1–17 years	11 (4.3)	6 (2.2)
18–24 years	1 (0.4)	8 (2.9)
25–44 years	12 (4.7)	10 (3.6)
45–64 years	33 (12.9)	36 (13.1)
65–84 years	142 (55.5)	135 (49.1)
> 84 years	55 (21.5)	75 (27.3)
Women, n (%)	131 (51.2)	138 (50.2)
Residence before the index admission, n (%)		
Home	230 (89.8)	246 (89.5)
Nursing care facility	14 (5.5)	26 (9.5)
Other hospitals	12 (4.7)	3 (1.1)
Past medical history, n (%)		
Ischemic heart disease	16 (6.3)	15 (5.5)
Stroke	34 (13.3)	54 (19.6)
Dementia	21 (8.2)	36 (13.1)
Liver cirrhosis	8 (3.1)	11 (4.0)
Diabetes mellitus	47 (18.4)	65 (23.6)
Dialysis	0 (0.0)	0 (0.0)
Immunosuppression drug use, n (%)	23 (9.0)	23 (8.4)
Location in hospitals, n (%)		
Ward	247 (96.5)	265 (96.4)
Critical care unit	9 (3.5)	10 (3.6)
Central line in place on survey date, n (%)	17 (6.6)	8 (2.9)
Peripheral line in place on survey date, n (%)	88 (34.4)	90 (32.7)
Urinary catheter in place on survey date, n (%)	33 (12.9)	39 (14.2)
Intubated or tracheal tube in place on survey date, n (%)	2 (0.8)	2 (0.7)
Drainage tube in place on survey date, n (%)	6 (2.3)	10 (3.6)
Median days to survey date from admission (IQR)	9 (3–22)	9 (3–20)

In the present survey, a total of 87 antimicrobial drugs were given to 79 patients (Table 2). The prevalence of any antimicrobial drug use was 28.7% (95% confidence interval (CI) 23.4% to 34.1%). The proportion of patients who received any antimicrobial drugs did not change from the previous survey to the present survey (28.5% versus 28.7%; odds ratio (OR) 0.99, 95% CI 0.68 to 1.44). Ceftriaxone accounted for more than one-fourth of all antimicrobial drugs used in the present survey. The proportion of ceftriaxone in all antimicrobial drugs significantly increased from the former survey to the present survey (13.9% versus 28.7%; OR 0.40, 95% CI 0.18 to 0.88). The proportion of cefazolin in all antimicrobial drugs was 0.0%, although cefazolin was most the frequently used antimicrobial drug in the previous survey.

**Table 2:** Groups of antimicrobial drugs given to surveyed patients according to the survey year

Type^a^	August 2018^b^	August 2019^b^
All antimicrobial drugs	79 (100.0)	87 (100.0)
First-generation cephalosporins	20 (25.3)	4 (4.6)
Cefazolin	19 (24.1)	0 (0.0)
Cefalexin	1 (1.3)	4 (4.6)
Penicillin combinations	16 (20.3)	10 (11.5)
Piperacillin–tazobactam	3 (3.8)	4 (4.6)
Ampicillin–sulbactam	12 (15.2)	6 (6.9)
Amoxicillin–clavulanate	1 (1.3)	0 (0.0)
Third-generation cephalosporins	15 (19.0)	33 (37.9)
Ceftriaxone	11 (13.9)	25 (28.7)
Cefotaxime	0 (0.0)	3 (3.4)
Cefoperazone–sulbactam	0 (0.0)	1 (1.1)
Cefcapene pivoxil	1 (1.3)	1 (1.1)
Ceftazidime	2 (2.5)	0 (0.0)
Cefditoren pivoxil	1 (1.3)	3 (3.4)
Second-generation cephalosporins	12 (15.2)	12 (13.8)
Cefmetazole	9 (11.4)	7 (8.0)
Cefotiam	3 (3.8)	0 (0.0)
Cefaclor	0 (0.0)	0 (0.0)
Flomoxef	0 (0.0)	5 (5.7)
Extended-spectrum penicillins	5 (6.3)	7 (8.0)
Piperacillin	1 (1.3)	0 (0.0)
Ampicillin	1 (1.3)	6 (6.9)
Amoxicillin	3 (3.8)	1 (1.1)
Sulfonamide and trimethoprim combinations	4 (5.1)	1 (1.1)
Trimethoprim–sulfamethoxazole	4 (5.1)	1 (1.1)
Macrolides	3 (3.8)	0 (0.0)
Erythromycin	1 (1.3)	0 (0.0)
Clarithromycin	1 (1.3)	0 (0.0)
Azithromycin	1 (1.3)	0 (0.0)
Tetracyclines	2 (2.5)	1 (1.1)
Minocyclin	2 (2.5)	1 (1.1)
Glycopeptide (parenteral)	1 (1.3)	2 (2.3)
Vancomycin	1 (1.3)	2 (2.3)
Intestinal anti-infectives	0 (0.0)	5 (5.7)
Metronidazole	0 (0.0)	3 (3.4)
Rifaximin	0 (0.0)	2 (2.3)
Fourth-generation cephalosporins	0 (0.0)	3 (3.4)
Cefozopran	0 (0.0)	3 (3.4)
Fluoroquinolones	0 (0.0)	3 (3.4)
Levofloxacin	0 (0.0)	3 (3.4)
Lincosamides	0 (0.0)	2 (2.3)
Clindamycin	0 (0.0)	2 (2.3)
Nucleoside and nucleotide antivirals	0 (0.0)	2 (2.3)
Acyclovir	0 (0.0)	1 (1.1)
Valaciclovir	0 (0.0)	1 (1.1)
Aminoglycosides	0 (0.0)	1 (1.1)
Gentamicin	0 (0.0)	1 (1.1)
Antimycotics	0 (0.0)	1 (1.1)
Amphotericin B	0 (0.0)	1 (1.1)

^a^Antimicrobial drugs were classified and reported based on the World Health Organization Anatomical Therapeutic Chemical fifth-level classification. Antimicrobial drugs were listed in order of most frequent use in the 2018 survey

^b^Data are expressed as numbers (percentages)

Of the 87 antimicrobial drugs used in the present survey, 51 (58.6%) and 33 (37.9%) were used for treatment and surgical prophylaxis, respectively (see Additional file 1). Of the 51 antimicrobial drugs used for treatment in the present survey, 21 (41.2%) were judged as appropriate antibiotic use (Table 3). The proportion of appropriate antimicrobial drug use in all antimicrobial therapy tended to be lower in the present survey than in the former survey (41.2% versus 60.6%; OR 2.18, 95% CI 0.96 to 5.00). The most common reason for the inappropriateness of antimicrobial therapy was an unnecessary prescription of a broader spectrum of antimicrobial drugs. The proportion of antimicrobial drugs that were judged to be inappropriately used for their spectrum activity significantly increased in the present survey from the previous survey (37.3% versus 4.7%; OR 10.93, 95% CI 2.33 to 51.19).

**Table 3:** The appropriateness of antimicrobial therapy according to survey year

	August 2018 (n = 43)^a^	August 2019 (n = 51)^a^	*p*-value^b^
Appropriate^c^	26 (60.6)	21 (41.2)	0.06
Inappropriate	17 (39.4)	30 (58.8)	0.06
Reasons for inappropriateness^d^			
No infection	7 (16.3)	5 (9.8)	0.35
Inappropriate dose or timing	9 (20.9)	6 (11.8)	0.14
Inappropriate duration	0 (0.0)	2 (3.9)	0.50
Inappropriate choice	0 (0.0)	4 (7.8)	0.25
Inappropriate spectrum^e^	2 (4.7)	19 (37.3)	0.002

^a^Data are shown as numbers (percentages)

^b^Comparison between the 2018 survey and the 2019 survey was performed using Fisher’s exact test

^c^The appropriateness of antimicrobial therapy was determined by members of the infection control team of each hospital based on the results of microbial tests at the time of the survey, antibiograms of each hospital, and the Infectious Diseases Society of America Practical Guidelines

^d^One antimicrobial drug could be given for more than one reason

^e^Antimicrobial therapy was judged to be inappropriate if there were alternative antimicrobial drugs that were equally effective and had narrower spectrum activity

### Discussion

The present study found that the prevalence of antimicrobial drug use in hospitalized patients did not change from the 2018 survey to the 2019 survey in a Japanese hospital with no supply of cefazolin since its shortage. The prevalence of antimicrobial drug use in the present survey is similar to those of European studies [6, 7, 9, 15] and recent Japanese studies [5, 13, 14] reporting that approximately one-third of hospitalized patients were receiving antimicrobial drugs. However, the proportion of ceftriaxone in all antimicrobial drugs used for hospitalized patients significantly increased from the 2018 survey to the 2019 survey, while cefazolin, which was most frequently used in the 2018 survey [14], was not used at all in the 2019 survey. In past studies investigating the trend of inpatient antimicrobial use [28–30], no studies reported a considerable change in an individual antimicrobial drug within a year similar to our study. A shortage of cefazolin since the beginning of 2019 in Japan is assumed to have increased the use of ceftriaxone as an alternative to cefazolin for hospitalized patients. However, whether the shortage of cefazolin was truly a major cause of the increased use of ceftriaxone is uncertain because causality between the two events cannot be proven by our study design. Further studies to investigate the change in antimicrobial drug use before and after the cefazolin shortage in other Japanese hospitals with or without supply of cefazolin are needed.

The present survey found that approximately three-fifths of antimicrobial therapies were judged to be inappropriate in a Japanese hospital with no supply of cefazolin since the beginning of 2019. Moreover, the appropriateness of inpatient antimicrobial therapy in the present survey tended to be worse than that of the previous survey [14], although it did not reach statistical significance. Given that unnecessary prescription of a broader spectrum of antimicrobial drugs significantly increased from the previous survey to the present survey, unnecessary prescription of a broader spectrum of antimicrobial drugs is presumed to be a major cause for the decline in the proportion of appropriate antimicrobial therapy from August 2018 to August 2019. The proportion of inappropriate antimicrobial therapy reported in the present survey was higher than that of past point prevalence surveys conducted since 2000 [6, 12, 14, 15, 18, 19, 31]. Given that some past similar studies did not evaluate the appropriateness for the spectrum activity of antimicrobial therapy [6, 12, 15, 18], the difference in methods used to evaluate the appropriateness of antimicrobial therapy might affect our findings. In fact, a recent retrospective study [32] investigating the appropriateness of antimicrobial therapy for uncomplicated cellulitis also reported that the proportion of inappropriate broad spectrum antimicrobial drug use was high, as in our study.

The cause of the decline in the appropriateness of inpatient antimicrobial therapy in the present study is uncertain. One repeated prevalence survey to evaluate the appropriateness of inpatient antimicrobial drug use also reported a considerable change in the appropriateness of antimicrobial therapy within a year in some surveyed hospitals [19]. Given that cefazolin was the antimicrobial drug used frequently and appropriately for treatment in the 2018 survey [14] and that cefazolin was not used at all in the 2019 survey, our findings implicate that a shortage of cefazolin since the beginning of 2019 may worsen the appropriateness of inpatient antimicrobial therapy in a Japanese hospital with no supply of cefazolin. Nonetheless, our study did not evaluate the change in the appropriateness of antimicrobial therapy before and after a shortage of cefazolin in Japanese hospitals that were not affected by a cefazolin shortage. Moreover, many confounding factors that could affect outcomes cannot be excluded because our study design was a longitudinal cross-sectional study. Thus, further multicenter studies to investigate the change in appropriateness of antimicrobial therapy before and after a cefazolin shortage in Japanese hospitals with or without supply of cefazolin are needed.

### Conclusions

The prevalence of inappropriate antimicrobial therapy was high and tended to increase from August 2018 to August 2019 in a Japanese hospital with no supply of cefazolin since March 2019. The shortage of cefazolin might lead to inappropriate use of other antimicrobial drugs.

## Limitations

First, our study was a single center study. Therefore, our results cannot be generalized to other Japanese hospitals. Second, this was not a randomized controlled design. Moreover, our study had no control groups. Therefore, the association between the shortage of cefazolin and inappropriate use of antimicrobial drugs with a broader spectrum in the present study should be interpreted cautiously.

## Supplementary information

**Additional file 1: Table S1.** Prevalence of antimicrobial drug use according to different specialties according to the survey year. **Table S2.** The reasons for antimicrobial drug use according to survey year. **Table S3.** Number and type of antimicrobial drugs given to treat infections according to the survey year. ^a^Data are expressed as numbers (percentages). **Table S4.** Infection sites for which patients received antimicrobial therapy according to the survey year. ^a^Antimicrobial drugs and patients could be given for more than one infection site. ^b^Data are expressed as numbers (percentages). **Table S5.** Appropriateness of the antimicrobial drugs used for treatment according to the location in the hospital, specialty, type of antimicrobial drug, and type of infection. ^a^Data are expressed as numbers (percentages). **Table S6.** Type and duration of antimicrobial drug used for surgical prophylaxis according to the survey year. ^a^Data are expressed as numbers (percentages). ^b^A total of 28 and 33 antimicrobial drugs were given to 28 and 32 patients for surgical prophylaxis in August 2018 and 2019, respectively.

## Data Availability

All data generated or analyzed during this study are included in this published article and its additional file.

## Abbreviations

CI: confidence interval
IQR: interquartile range
NA: not available
OR: odds ratio
SD: standard deviation
