# Supplementary material for: A trend in prevalence of antimicrobial use and appropriateness of antimicrobial therapy in an acute care hospital from 2018 to 2019: repeated prevalence surveys in Japan
Source: BMC Res Notes. 2019 Dec 18;12:811. doi: 10.1186/s13104-019-4849-0 (PMC6918669; doi:10.1186/s13104-019-4849-0)
Supplement: Supplementary file 1 — Additional file 1: Table S1. Prevalence of antimicrobial drug use according to different specialties according to the survey year. Table S2. The reasons for antimicrobial drug use according to survey year. Table S3. Number and type of antimicrobial drugs given to treat infections according to the survey year. aData are expressed as numbers (percentages). Table S4. Infection sites for which patients received antimicrobial therapy according to the survey year. aAntimicrobial drugs and patients could be given for more than one infection site. bData are expressed as numbers (percentages). Table S5. Appropriateness of the antimicrobial drugs used for treatment according to the location in the hospital, specialty, type of antimicrobial drug, and type of infection. aData are expressed as numbers (percentages). Table S6. Type and duration of antimicrobial drug used for surgical prophylaxis according to the survey year. aData are expressed as numbers (percentages). bA total of 28 and 33 antimicrobial drugs were given to 28 and 32 patients for surgical prophylaxis in August 2018 and 2019, respectively. [file 13104_2019_4849_MOESM1_ESM.pdf]

Table S1. Prevalence of Antimicrobial Drug Use According to Different Specialties According to the Survey Year.

**Specialty**	**Number of patients**	**Prevalence, %**
Total		
August 2018	256	28.5
August 2019	275	28.7
General surgery		
August 2018	53	30.2
August 2019	35	28.6
Orthopaedic surgery		
August 2018	46	17.4
August 2019	42	35.7
Neurosurgery		
August 2018	23	30.4
August 2019	34	23.5
Gynaecology		
August 2018	4	25.0
August 2019	2	50.0
Oral and maxillofacial surgery		
August 2018	13	76.9
August 2019	17	41.2
Otorhinolaryngology		
August 2018	5	60.0
August 2019	8	87.5
Ophthalmology		
August 2018	0	NA
August 2019	3	100.0
Urology		
August 2018	13	23.1
August 2019	14	21.4
Dermatology		
August 2018	4	25.0
August 2019	2	50.0
Internal medicine		
August 2018	90	23.3

August 2019	108	19.4
Paediatric surgery		
August 2018	0	NA
August 2019	2	0.0
Paediatrics		
August 2018	5	60.0
August 2019	8	37.5

NA, not available.

Table S2. The Reasons for Antimicrobial Drug Use According to Survey Year.

**Reasons**	**August 2018 (n = 79)**	**August 2019 (n = 87)**
Treatment for infection, n (%)	43 (54.4)	51 (58.6)
Prophylaxis, n (%)		
Surgical	28 (35.4)	33 (37.9)
Medical	5 (6.3)	1 (1.1)
Noninfectious reasons, n (%)	1 (1.3)	2 (2.3)
Unknown reasons, n (%)	2 (2.5)	0 (0.0)

Table S3. Number and Type of Antimicrobial Drugs Given to Treat Infections According to the Survey Year.

**Type**	**August 2018[a]**	**August 2019[a]**
Total antimicrobial drugs	43 (100.0)	51 (100.0)
Ceftriaxone	9 (20.9)	15 (29.4)
Cefmetazole	7 (16.3)	3 (5.9)
Cefazolin	6 (14.0)	0 (0.0)
Ampicillin-sulbactam	5 (11.6)	3 (5.9)
Piperacillin-tazobactam	3 (7.0)	4 (7.8)
Ceftazidime	2 (4.7)	0 (0.0)
Amoxicillin	2 (4.7)	1 (2.0)
Ampicillin	1 (2.3)	1 (2.0)
Amoxicillin-clavulanate	1 (2.3)	0 (0.0)
Azithromycin	1 (2.3)	0 (0.0)
Cefditoren pivoxil	1 (2.3)	3 (5.9)
Minocycline	1 (2.3)	1 (2.0)
Piperacillin	1 (2.3)	0 (0.0)
Vancomycin (parenteral)	1 (2.3)	2 (3.9)
Trimethoprim-sulfamethoxazole	1 (2.3)	1 (2.0)
Cefalexin	1 (2.3)	0 (0.0)
Cefozopran	0 (0.0)	3 (5.9)
Cefotaxime	0 (0.0)	3 (5.9)
Metronidazole (parenteral)	0 (0.0)	2 (3.9)
Levofloxacin	0 (0.0)	2 (3.9)
Clindamycin	0 (0.0)	2 (3.9)
Acyclovir	0 (0.0)	1 (2.0)
Amphotericin B (enteral)	0 (0.0)	1 (2.0)
Gentamicin	0 (0.0)	1 (2.0)
Metronidazole (enteral)	0 (0.0)	1 (2.0)
Valacyclovir	0 (0.0)	1 (2.0)

[a]Data are expressed as numbers (percentages).

Table S4. Infection Sites for Which Patients Received Antimicrobial Therapy According to the Survey Year.

Infection site[a]	August 2018[b]		August 2019[b]	
	Number of drugs, n (%) (n = 43)	Number of patients, n (%) (n = 40)	Number of drugs, n (%) (n = 51)	Number of patients, n (%) (n = 44)
Hepatobiliary system	10 (23.3)	10 (25.0)	2 (3.9)	2 (4.5)
Bloodstream	9 (20.9)	9 (22.5)	6 (11.8)	6 (13.6)
Urinary tract	8 (18.6)	7 (17.5)	9 (17.6)	9 (20.5)
Skin and soft tissue	7 (16.3)	6 (15.0)	5 (9.8)	5 (11.4)
Eye, ear, nose, throat, or mouth	5 (11.6)	5 (12.5)	4 (7.8)	3 (6.8)
Lower respiratory tract	4 (9.3)	3 (7.5)	9 (17.6)	9 (20.5)
Intra-abdominal	3 (7.0)	3 (7.5)	0 (0.0)	0 (0.0)
Gastrointestinal tract	3 (7.0)	3 (7.5)	6 (11.8)	4 (9.1)
Empirical/undetermined	1 (2.3)	1 (2.5)	1 (2.0)	1 (2.3)
Bone and joint	2 (4.7)	2 (5.0)	6 (11.8)	5 (11.4)
Central nervous system	2 (4.7)	2 (5.0)	5 (9.8)	2 (4.5)
Others	0 (0.0)	0 (0.0)	1 (2.0)	1 (2.3)

[a]Antimicrobial drugs and patients could be given for more than one infection site.

[b]Data are expressed as numbers (percentages).

Table S5. Appropriateness of the Antimicrobial Drugs Used for Treatment According to the Location in the Hospital, Specialty, Type of Antimicrobial Drug, and Type of Infection.

	August 2018[a]		August 2019[a]	
	**Total number of uses**	**Number of inappropriate uses**	**Total number of uses**	**Number of inappropriate uses**
Location in hospital				
Ward	41	15 (36.6)	51	30 (58.8)
Critical care unit	2	2 (100.0)	0	NA
Specialty				
Internal medicine	17	0 (0.0)	19	8 (42.1)
General surgery	8	6 (75.0)	4	3 (75.0)
Neurosurgery	4	4 (100.0)	7	5 (71.4)
Orthopaedic surgery	1	1 (100.0)	6	4 (66.7)
Otorhinolaryngology	1	0 (0.0)	5	2 (40.0)
Others	12	6 (50.0)	10	8 (80.0)
Type of antimicrobial drug				
Ceftriaxone	9	4 (44.4)	15	7 (46.7)
Cefmetazole	7	2 (28.6)	3	1 (33.3)
Cefazolin	6	1 (16.7)	0	NA
Ampicillin-sulbactam	5	4 (80.0)	3	2 (66.7)
Others	16	6 (37.5)	30	20 (66.7)
Site of infection				
Hepatobiliary system	10	2 (20.0)	2	0 (0.0)
Bloodstream	9	1 (11.1)	5	2 (40.0)
Urinary tract	8	2 (25.0)	9	6 (66.7)
Skin and soft issue	5	2 (40.0)	5	5 (100.0)
Lower respiratory tract	4	2 (50.0)	8	5 (62.5)
Bone and joint	2	0 (0.0)	6	4 (66.7)
Gastrointestinal tract	2	2 (100.0)	6	1 (16.7)

[a]Data are expressed as numbers (percentages).

NA, not available.

Table S6. Type and Duration of Antimicrobial Drug Used for Surgical Prophylaxis According to the Survey Year.

	August 2018[a] (n = 28)	August 2019[a] (n = 33)
Duration		
Mean days (SD)	2.5 (1.3)	1.8 (1.1)
Median days (IQR)	2 (2 – 3)	2 (1 – 2)
One day	4 (14.3)	16 (48.5)
Two days	11 (39.3)	11 (33.3)
More than two days	13 (46.4)	6 (18.2)
Type of antimicrobial drugs[b]		
Cefazolin	13 (46.4)	0 (0.0)
Ampicillin-sulbactam	7 (25.0)	3 (9.1)
Cefotiam	3 (10.7)	0 (0.0)
Cefmetazole	2 (7.1)	4 (12.1)
Ceftriaxone	1 (3.6)	10 (30.3)
Cefcapene pivoxil	1 (3.6)	1 (3.0)
Amoxicillin	1 (3.6)	0 (0.0)
Flomoxef	0 (0.0)	5 (15.2)
Ampicillin	0 (0.0)	5 (15.2)
Cefalexin	0 (0.0)	4 (12.1)
Cefoperazone-sulbactam	0 (0.0)	1 (3.0)

[a]Data are expressed as numbers (percentages).

[b]A total of 28 and 33 antimicrobial drugs were given to 28 and 32 patients for surgical prophylaxis in August 2018 and 2019, respectively.

IQR, interquartile range; SD, standard deviation.
